# Interpregnancy interval and maternal and neonatal morbidity: a nationwide cohort study

**DOI:** 10.1038/s41598-022-22290-1

**Published:** 2022-10-18

**Authors:** Hanna Mühlrad, Evelina Björkegren, Philip Haraldson, Nina Bohm-Starke, Helena Kopp Kallner, Sophia Brismar Wendel

**Affiliations:** 1 Department of Clinical Sciences, Karolinska Institutet, Danderyd Hospital, Stockholm, Sweden; 2 Department of Women’s Health, Danderyd Hospital, Stockholm, Sweden; 3 The Institute for Evaluation of Labor Market and Education Policy, Uppsala, Sweden; 4 Department of Economics, Stockholm University, Stockholm, Sweden

**Keywords:** Medical research, Epidemiology

## Abstract

This study aimed to assess the association between interpregnancy interval (IPI)—the time from childbirth to conception of the next pregnancy—and maternal and neonatal morbidity. The World Health Organization (WHO) currently recommends an IPI of at least 24 months after a live birth to reduce adverse birth outcomes. However, assessing the relationship between IPI and perinatal outcome is complicated by confounding factors. We conducted a nationwide population-based cohort study using Swedish registry data, allowing for adjustment of maternal characteristics and health at first birth. The study population consisted of all women with a singleton, live, and vaginal first birth with a second singleton birth within five years during 1997–2017, covering 327,912 women and 655,824 neonates. IPI was grouped into six-month intervals with 24–29 months as the reference. The association between IPI and morbidity was examined using multivariate logistic regression. For women having a vaginal delivery at their first birth, intervals < 24–29 months were associated with decreased maternal morbidity and unaffected neonatal morbidity. Intervals > 24–29 months were associated with increased maternal and neonatal morbidity. Our findings question the relevance of WHO’s recommendation of an IPI of at least 24 months in a high-income country.

## Introduction

The interpregnancy interval (IPI), i.e. the time from childbirth to conception of the next pregnancy, may affect health at childbirth for the mother and neonate. A short IPI has been linked to adverse pregnancy outcomes^1,2^, most notably maternal mortality^3^, but also elevated risks of stillbirth and birth defects^4,5^. A short IPI has also been associated with preterm birth^1,2,6^, small for gestational age/low birthweight at term^1,7^, and preterm prelabor rupture of membranes^8,9^. Other observed associated risks are maternal nutrient deficiencies, especially in lactating and malnourished women^7,10–12^. As a consequence, the World Health Organization (WHO) has issued a recommendation suggesting an IPI of at least 24 months after a live birth and 6 months after an early pregnancy loss^13^.

On the other hand, a long IPI has been associated with increased risk of fetal death^14^, preterm birth^15^, small for gestational age/low birthweight^16^, and pre-eclampsia^17^. In addition, a long IPI may be associated with the loss of adaptive benefits in the woman from a previous birth but can also entail effects of aging, change of partner, and change in socioeconomic status^12,18–21^.

Moreover, many of the studies behind the recommended IPI from WHO emanate from low- and middle-income countries^13^, while the effects of IPI in high-resource countries are less clear. As an example, a cohort study with data from USA, Australia, Finland, and Norway showed that the association between IPI and preterm birth was modified by whether or not the previous pregnancy was preterm^22^. Due to confounding factors, assessing the association between IPI and perinatal outcome is challenging. These factors include underlying health of the woman, previous induced and spontaneous abortions, and other health behavior and preferences^23^.

While the literature on the impact of short IPIs for maternal and neonatal health is extensive, the impact of long IPIs and the causal link has yet to be established and the mechanisms by which IPI affects specific birth outcomes are not well understood^23^. The aim of this study was to examine the association between IPI and maternal and neonatal morbidity using nationwide health registers and population statistics in Sweden. We also examined the importance of confounding factors in explaining the relationship between IPI and maternal and neonatal outcomes.

## Methods

### Data sources

Information on maternal and neonatal pregnancy outcomes was retrieved from the Swedish Medical Birth Register. These data, provided by the Swedish National Board of Health and Welfare, contain extensive prospectively collected information on pregnancy, delivery, and postpartum conditions for pregnancies beyond 22 weeks of gestational age, for both stillbirths and livebirths.

The link between each woman and child was retrieved from the Multi-Generation Register (Statistics Sweden). Information on previous hospitalizations came from the National Patient Register (Swedish National Board of Health and Welfare). Information on maternal educational attainment and income from sickness benefits were retrieved from the Longitudinal Integration Database for Health Insurance and Labor Market Studies (Statistics Sweden).

### Sample selection

**Figure 1:**
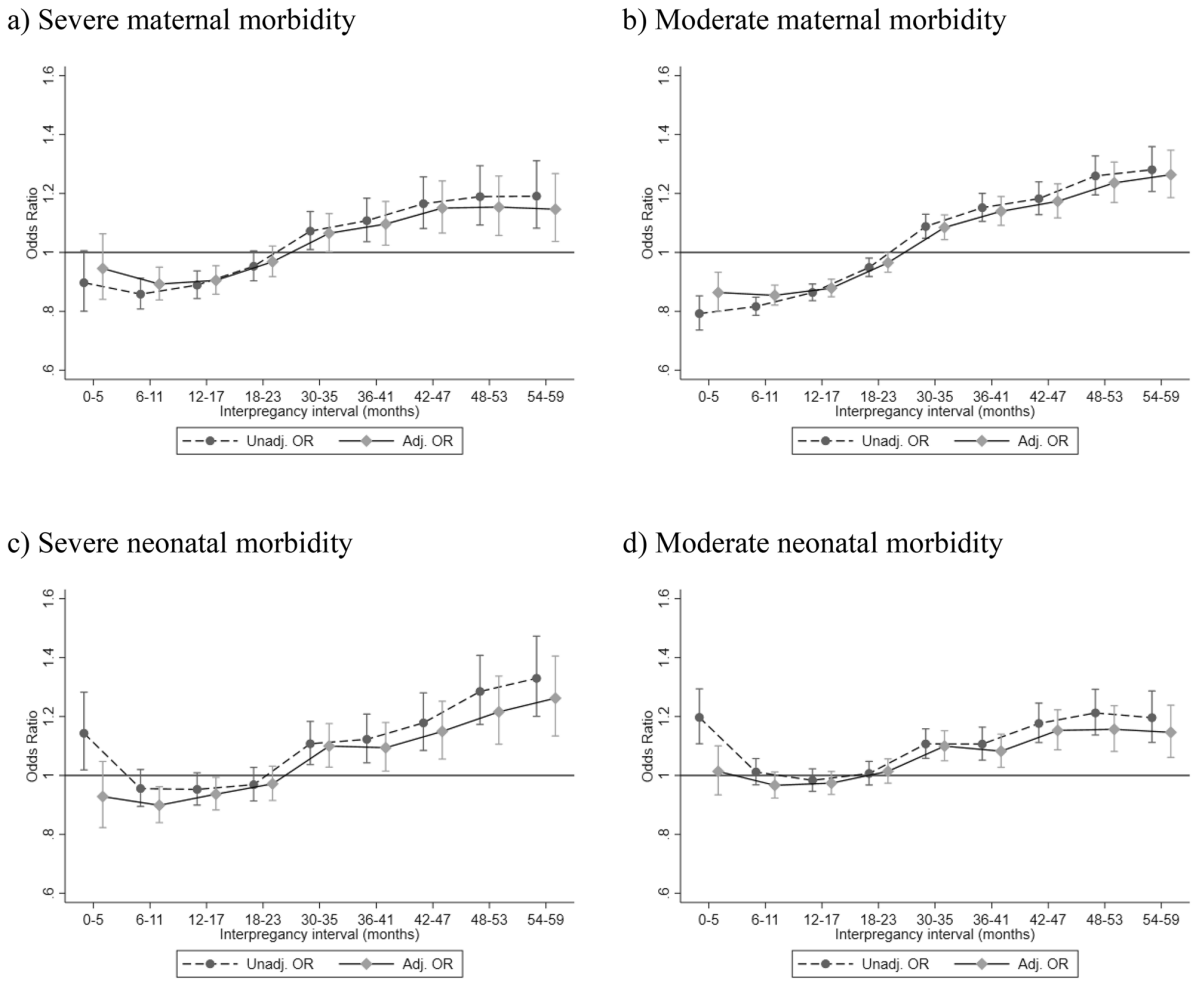
Association between interpregnancy interval and maternal and neonatal morbidity. Unadj: unadjusted, Adj: adjusted, OR: odds ratio. The odds ratios are presented with 95% confidence intervals marked by the whiskers. 24–29 months are the reference interval. The reference (1) is marked with a solid line.Values below 1 represent a decreased risk and values above 1 represent an increased risk. Adjustment was made for educational attainment, body mass index, age, born in Sweden or not, smoking, in vitro fertilization, less than 5 prenatal visits, sickness benefits or hospitalization prior to the first birth, and severe and moderate maternal and neonatal morbidity in the first birth.

We retrieved a sample based on all births during 1997–2017 identified via the Swedish Medical Birth Register and the Multi-Generation Register (Supporting information, Fig. S1. Flow chart). The sample was restricted to women giving birth to their first and second child during 1997–2017, and to those having their second child within 5 years from the first child. The sample was further restricted to women having a single and liveborn child, delivered vaginally, at the first birth. Cesarean first births were excluded since they may already carry a burden of disease. Women without missing information on pertinent covariates (age, parity, BMI, educational attainment, smoking, country of birth, prenatal visits, income, and hospital identifier) were included (Fig. 1). Our final sample contained 327,912 unique women and 655,824 neonates.

### Exposure and classification of covariates

The exposure IPI is defined from birth to conception and was computed using the time elapsed between the first and the second birth date after subtracting gestational age in weeks of the second child. The IPI was grouped into six-month intervals (0–5, 6–11, 12–17, 18–23, 24–29, 30–35, 36–41, 42–47, 48–53, 54–59 months) up to five years after the first birth. The distribution of IPIs is presented in Supporting information (Fig. S2) and showed that the most common IPI is 12–17 months followed by 18–23 and 24–29 months. Maternal characteristics included the following variables from the second birth; educational attainment (primary, secondary, tertiary), body mass index (BMI; ≤ 25.0, 25.1–30.0, > 30.0 kg/m^2^) at the first visit in antenatal health care, age (< 25, 25–29, 30–34, 35–39, > 40 years), born in Sweden (yes/no), smoking tobacco in early pregnancy (yes/no), in vitro fertilization (IVF, yes/no), and less than 5 prenatal visits (yes/no). These characteristics were included in the multivariate logistic regression analyses. In addition, we controlled for receiving sickness benefits and hospitalization prior the first birth, and severe and moderate maternal and neonatal morbidity in the first birth (defined below).

### Outcome measures

Maternal and neonatal morbidity were defined using medical diagnoses and surgical procedures according to the International Classification of Diseases—10th Revision (ICD-10) Sweden and the classification of care procedures (Swedish “KVÅ codes”). A core outcome set has not yet been defined for IPI exposure. Composite outcome sets for severe and moderate morbidity were constructed for women and neonates, respectively. A binary indicator was constructed for each class of morbidity, which was set to 1 for individuals with at least one case of morbidity, and 0 for the remainder. Each component of the composite index for, severe and moderate, maternal and neonatal morbidity is presented in Tables 2 and 3, respectively. More detailed information regarding the ICD-codes used for definition morbidity are provided in Supporting information, Tables S1 and S2.

### Statistical analyses

Differences in maternal characteristics across IPIs were examined using Kruskal–Wallis tests. Characteristics that differed across IPIs based on this test were considered potential confounders and adjusted for in the regression analysis. The associations between IPI and maternal and neonatal morbidity were assessed using logistic regression, calculating the unadjusted and adjusted odds ratio (OR and aOR), using 24–29 months as reference. We constructed the IPIs as indicator variables to flexibly allow the effect to vary across the distribution and focused on 6-month intervals up to five years after the first birth. An IPI less than 24 months is termed a short IPI, while more than 29 months is termed a long IPI.

In the adjusted analyses, we controlled for a full set of confounders: educational attainment (primary, secondary, tertiary), body mass index (BMI; ≤ 25.0, 25.1–30.0, > 30.0 kg/m^2^) at the first visit in antenatal health care, age (< 25, 25–29, 30–34, 35–39, > 40 years), born in Sweden (yes/no), smoking tobacco in early pregnancy (yes/no), in vitro fertilization (IVF, yes/no), less than 5 prenatal visits (yes/no) in the second birth, and receiving sickness benefits or hospitalization prior the first birth, and severe and moderate maternal and neonatal morbidity in the first birth. To explore the importance of maternal selection behind the results, we examined how estimates were affected by the inclusion of maternal covariates and outcomes at first birth. We postulated that selection based on observables provides information on selection on unobservable characteristics^24^. Finally, we examined sensitivity to the inclusion of women with missing information on covariates by estimating the same unadjusted model for the women with missing information on maternal characteristics. Data analyses were conducted using STATA 14.0 (STATACorp, College Station, Texas, USA). This study is reported according to the Strengthening the Reporting of Observational Studies in Epidemiology (STROBE) guideline^25^.

### Ethics approval

The study was conducted after it was approved by the Swedish Ethical Review Authority on November 25, 2019 (2019-05125) and all methods were performed in accordance with the relevant guidelines and regulations of the Swedish Ethical Review Authority. According to Swedish law, information from these registers is collected and available for research after permission from the Swedish Ethical Review Authority. The authors are willing to assist anyone who wishes to access this data set. In protection of the integrity and anonymity of the individuals and legal entities, data are pseudomized. Individual consent is not collected for participation in these registers, nor is is it collected for research conducted on the information obtained in the registers. Thus, informed consent on individual level is waived by the Swedish Ethical Review Authority (“Etikprövningsmyndigheten”, https://etikprovningsmyndigheten.se/).

## Results

### Differences in maternal characteristics across IPI

Maternal characteristics differed across IPIs (Table 1). Women with short IPIs had lower education attainment, higher BMI, younger age, were more likely to be born outside of Sweden, to be smoking in early pregnancy, receiving sickness benefits, and being hospitalized prior to the first birth, compared with women with longer IPIs (Table 1). While maternal morbidity at the first birth was less prevalent, neonatal morbidity at the first birth was more prevalent in women with short IPIs, compared with women with long IPIs (Table 1).

**Table 1:** Maternal characteristics across interpregnancy intervals.

	< 6 months		6–11 months		12–17 months		18–23 months		≥ 24 months	
	n	%	n	%	n	%	n	%	n	%
**Education**										
Primary	1605	21.5	4469	10.8	4868	7.0	3898	6.3	11,554	7.8
Secondary	3527	47.3	17,930	43.5	28,139	40.3	23,663	38.2	61,905	42.0
Tertiary	2318	31.1	18,826	45.7	36,772	52.7	34,376	55.5	74,062	50.2
**BMI (kg/m**^**2**^**)**										
≤ 25.0	3694	49.6	25,313	61.4	45,214	64.8	41,001	66.2	92,175	62.5
25.1–30.0	2273	30.5	10,302	25.0	16,290	23.3	14,074	22.7	36,401	24.7
> 30.0	1332	17.9	4528	11.0	6440	9.2	5306	8.6	15,865	10.8
**Age (years)**										
< 25	2349	31.5	7046	17.1	7601	10.9	5566	9.0	10,571	7.2
25–29	2705	36.3	14,643	35.6	23,192	33.3	19,286	31.2	43,328	29.4
30–34	1707	22.9	14,016	34.0	28,376	40.7	27,309	44.1	63,557	43.1
35–39	610	8.2	4922	11.9	9624	13.8	8843	14.3	26,608	18.0
≥ 40	79	1.1	582	1.4	964	1.4	912	1.5	3418	2.3
Born in Sweden	5180	69.5	34,367	83.4	61,466	88.1	55,082	88.9	125,868	85.3
Smoking	897	12.0	2372	5.8	2713	3.9	2493	4.0	8412	5.7
IVF	17	0.2	227	0.6	865	1.2	864	1.4	3384	2.3
< 5 Prenatal visits	6788	91.1	39,129	94.9	66,938	95.9	59,532	96.1	141,623	96.0
Sickness benefits*	1571	21.1	8527	20.7	13,547	19.4	10,791	17.4	25,878	17.5
Hospitalization*	402	5.4	1917	4.7	2731	3.9	2443	3.9	6525	4.4
**Morbidity in first birth**^†^										
Severe maternal	568	7.6	3730	9.0	6851	9.8	6378	10.3	15,146	10.3
Moderate maternal	2224	29.9	13,021	31.6	22,545	32.3	19,903	32.1	46,829	31.7
Severe neonatal	626	8.4	2785	6.8	4519	6.5	4198	6.8	10,190	6.9
Moderate neonatal	1059	14.2	5087	12.3	8403	12.0	7572	12.2	18,634	12.6
Missing^‡^	2274	23.4	8033	16.3	10,875	13.5	8786	12.4	19,683	11.8

*Prior to first birth.

^†^According to definitions in Supplementary tables S1 and S2.

^‡^Women with missing information on at least one covariate.

### Association between IPI and maternal and neonatal morbidity

Severe maternal morbidity at the second birth affected around 4.5% of all women and had the lowest prevalence at an IPI of 6–11 months (Table 2). Moderate maternal morbidity affected more than 12% of women and increased with increasing IPI (Table 2). For neonates at the second birth, composite severe and moderate morbidity was slightly U-shaped across IPIs with the lowest prevalence at 12–17 months (Table 3).

**Table 2:** Maternal morbidity in the second birth across interpregnancy intervals.

	< 6 months		6–11 months		12–17 months		18–23 months		≥ 24 months	
	n	%	n	%	n	%	n	%	n	%
**Severe morbidity**										
Composite severe morbidity*	350	4.70	1857	4.50	3250	4.66	3082	4.98	8262	5.60
Maternal death	0	0.00	1	0.00	1	0.00	5	0.01	4	0.00
Maternal sepsis	13	0.17	56	0.14	60	0.09	53	0.09	172	0.12
Eclampsia	2	0.03	5	0.01	8	0.01	9	0.01	28	0.02
Hysterectomy	0	0.00	2	0.00	5	0.01	3	0.00	11	0.01
Other major surgical intervention^**†**^	4	0.05	17	0.04	52	0.07	48	0.08	104	0.07
Transfusion	50	0.67	263	0.64	429	0.61	394	0.64	1002	0.68
Venous thromboembolism	1	0.01	7	0.02	12	0.02	16	0.03	27	0.02
Uterine rupture, obstetric injuries	57	0.77	276	0.67	489	0.70	456	0.74	1224	0.83
Intrapartum cesarean section	171	2.30	797	1.94	1345	1.93	1299	2.10	3846	2.61
3rd or 4th degree perineal lacerations	80	1.07	539	1.31	1002	1.44	948	1.53	2241	1.52
**Moderate morbidity**										
Composite moderate morbidity^‡^	916	12.30	5204	12.62	9253	13.26	8904	14.38	24,083	16.33
Postpartum hemorrhage > 1000 ml	342	4.59	1911	4.64	3253	4.66	3042	4.91	7803	5.29
Curettage	9	0.12	35	0.08	76	0.11	83	0.13	182	0.12
Cervical lacerations	15	0.20	67	0.16	111	0.16	92	0.15	241	0.16
Preeclampsia	119	1.60	596	1.45	1093	1.57	1075	1.74	3252	2.20
Diabetes, gestational	63	0.85	279	0.68	383	0.55	384	0.62	1177	0.80
Chorioamnionitis	5	0.07	20	0.05	35	0.05	30	0.05	102	0.07
Wound infection	2	0.03	13	0.03	15	0.02	6	0.01	32	0.02
Urinary tract infection	8	0.11	50	0.12	62	0.09	61	0.10	172	0.12
Endometritis	13	0.17	56	0.14	60	0.09	53	0.09	172	0.12
Episiotomy	129	1.73	854	2.07	1759	2.52	1668	2.69	4133	2.80
Forceps	6	0.08	26	0.06	46	0.07	36	0.06	112	0.08
Vacuum extraction	117	1.57	654	1.59	1231	1.76	1229	1.98	3380	2.29
Prelabor cesarean section	204	2.74	1229	2.98	2153	3.09	2211	3.57	6584	4.47

*All components of severe morbidity presented below.

^†^Uterine compression sutures (B-Lynch), uterine artery ligation or embolization, internal iliac artery ligation, intrauterine balloon tamponade.

^‡^All components of moderate morbidity presented below.

**Table 3:** Neonatal morbidity in the second birth across interpregnancy intervals.

	< 6 months		6–11 months		12–17 months		18–23 months		≥ 24 months	
	n	%	n	%	n	%	n	%	n	%
**Severe morbidity**										
Composite severe morbidity*	354	4.75	1650	4.00	2785	3.99	2512	4.06	6802	4.61
Stillbirth	13	0.17	87	0.21	127	0.18	141	0.23	331	0.22
Cardiorespiratory resuscitation	8	0.11	21	0.05	37	0.05	48	0.08	98	0.07
Mechanical ventilation	12	0.16	32	0.08	49	0.07	48	0.08	99	0.07
Hypoxic ischemic encephalopathy 2–3	0	0.00	13	0.03	31	0.04	21	0.03	80	0.05
Neonatal convulsions	2	0.03	25	0.06	61	0.09	46	0.07	126	0.09
Therapeutic hypothermia	0	0.00	0	0.00	5	0.01	2	0.00	7	0.00
Umbilical artery pH < 7.0	28	0.38	151	0.37	301	0.43	240	0.39	694	0.47
Apgar < 4 at 5 min	14	0.19	72	0.18	118	0.17	109	0.18	322	0.22
Meconium aspiration	139	1.87	597	1.45	1023	1.47	943	1.52	2594	1.76
Hypoglycemia	83	1.11	470	1.14	832	1.19	758	1.22	2111	1.43
Intracranial hemorrhage	8	0.11	25	0.06	22	0.03	27	0.04	78	0.05
Birth trauma^**†**^	46	0.62	247	0.60	373	0.53	313	0.51	773	0.52
Cerebral palsy	0	0.00	0	0.00	1	0.00	0	0.00	0	0.00
Sepsis	36	0.48	128	0.31	214	0.31	200	0.32	601	0.41
Pneumonia	7	0.09	55	0.13	83	0.12	84	0.14	195	0.13
Birthweight < 1500 g	34	0.46	91	0.22	139	0.20	144	0.23	411	0.28
Preterm birth < 32 weeks	85	1.14	238	0.58	365	0.52	376	0.61	1048	0.71
**Moderate morbidity**										
Composite moderate morbidity^‡^	835	11.21	3974	9.64	6556	9.40	5944	9.60	15,218	10.32
Apgar 4–6 at 5 min	25	0.34	133	0.33	247	0.36	248	0.40	691	0.47
Brachial plexus injury	22	0.30	80	0.19	130	0.19	117	0.19	266	0.18
Cephalo-/subgaleal hematoma	43	0.58	190	0.46	375	0.54	317	0.51	862	0.58
Jaundice	200	2.68	813	1.97	1308	1.87	1174	1.90	3217	2.18
Macrosomia	300	4.03	1874	4.55	3122	4.47	2903	4.69	6871	4.66
CPAP	40	0.54	130	0.32	232	0.33	206	0.33	551	0.37
Birthweight 1500–2500 g	127	1.70	522	1.27	809	1.16	783	1.26	2236	1.52
Preterm birth 32–36 weeks	272	3.65	1030	2.50	1576	2.26	1305	2.11	3631	2.46

*CPAP* continuous positive airway pressure.

*All components of severe morbidity presented below.

^†^Fractures, neurologic injury, retinal hemorrhage, or facial nerve palsy.

^‡^All components of moderate morbidity presented below.

The risk of severe maternal morbidity was lower for short IPIs and higher for intervals over 24–29 months, also after adjusting for maternal characteristics and outcomes at the first birth (Fig. 1 and Supporting information, Table S3). The lowest risk of severe maternal morbidity was seen at an IPI of 6–11 months (aOR 0.89, 95% CI 0.84–0.95). A similar pattern was seen for moderate maternal morbidity (Fig. 1, Table S3).

The risk of severe neonatal morbidity was unaffected for short IPIs and significantly higher for long IPIs compared with 24–29 months after adjusting for maternal characteristics and outcomes at the first birth (Fig. 1 and Supporting information, Table S4). The lowest risk of severe neonatal morbidity was seen at an IPI of 6–11 months (0.90, 95% CI 0.84–0.96). Likewise, the risk of moderate neonatal morbidity was unaffected for short IPIs and significantly higher for long IPIs (Fig. 1, Table S4).

The sensitivity analysis, comparing our estimates to those of women with missing information on maternal characteristics, suggested a similar result to our main findings (Supporting information, Fig. S3). The adjusted model for women with missing maternal characteristics was limited to adjusting for morbidity outcomes at the first birth. For women with missing information on maternal characteristics, a short IPI < 6 months was associated with higher risk for moderate neonatal morbidity (Fig. S3).

## Discussion

In this nationwide population-based cohort study, we found that IPIs shorter than 24 months were associated with a lower risk of maternal morbidity compared with 24–29 months. Short IPIs were not associated with higher risks of neonatal morbidity. IPIs longer than 24–29 months were associated with increased maternal and neonatal morbidity. We found the lowest risk of severe morbidity in both women and neonates with an IPI of 6–11 months. There was a difference between the unadjusted and adjusted results, which suggests that an adverse outcome at a second birth after a short IPI is rather reliant on the maternal characteristics and outcome at the first birth than the elapsed timed. This challenges the generalizability of the WHO recommended IPI of at least two years^13^.

The major strength of our study is the large sample size and the statistical power to evaluate associations with rare events. In addition, the Swedish national registers and linkage between them through the personal identification numbers enabled us to control for many indicators of pre-gestational maternal health, socioeconomic environment, education, and perinatal outcomes at the first birth. The Swedish Medical Birth Register has close to 100% coverage due to an easily accessible antenatal care program free of charge, specifically designed to identify women with need for hospital-based out- or inpatient care. All Swedish delivery units are situated in hospitals, free of charge, and are publicly reimbursed. There is virtually no unregistered private prenatal care, home births, or privately funded delivery units. Reporting to the Swedish Medical Birth Register is mandatory both for antenatal and hospital-based care. Despite this, some factors possibly affecting fertility and hereditary diseases were not available, such as ethnicity, cultural or religious views, or having a new partner. We aimed to adjust for ethnicity by country of birth outside Sweden and for reduced fertility by using information on IVF preceding the included birth as a marker. However, failed IVF-cycles are unfortunately not available in the registry and thus not controlled for.

Another limitation of the Swedish Medical Birth Register is that outcomes of pregnancies terminated before gestational week 22 are unavailable, which disabled adjustment for pregnancy loss before this week. Moreover, some variables are notoriously underreported and therefore not missing at random, such as maternal weight and smoking. We explored the impact of missing data in sensitivity analyses and found similar results between our sample and the population with missing data of covariates. However, when adjusted for outcomes of the first birth, the population with missing data of maternal covariates had an increased risk of moderate neonatal morbidity with a short IPI. This may be explained by missing data being overrepresented in variables considered to be stigmatizing, such as maternal weight or smoking. Thus, missing information in the antenatal medical records could be informative in itself and should evoke careful evaluation before advising the woman on birth spacing. Our study does not incorporate non-medical advantages of longer interpregnancy intervals such as stress, socio-economic consequences, time investments in partner and in the children^26^.

Our results are supported by previous studies from other high-resource settings like Canada, Australia, and Norway^27–29^, in which the authors made similar efforts to adjust for inherent risks. Our study adds more information on more possible confounders and on rare severe maternal and neonatal outcomes. We chose to construct and align one maternal and one neonatal composite outcome set, as one event in the woman could be at the expense of the neonate, and vice versa. For example, an intrapartum cesarean section (maternal morbidity) may prevent or cure fetal hypoxia and thereby reduce neonatal morbidity, while induction of labor preterm (neonatal morbidity) can prevent eclampsia (maternal morbidity). We also constructed two levels of severity to present both severe and less severe outcomes to nuance the risks.

We believe that our results are generalizable to many high-resource countries, but there are some specific circumstances to consider. Most women giving birth in Sweden are healthy and well-nourished. On the other hand, approximately one in four women giving birth in Sweden are not born in Sweden, contributing to a mixed risk population. Then again, the high availability and use of the Swedish maternal health care system may compensate for potential risks with short and long IPIs.

The clinical implication of our findings is that women in high-resource settings, with a normal previous birth, may choose to have children at a short time interval without increasing the risk of morbidity in the next pregnancy. However, the characteristics, behavior, and medical history of the woman may result in higher risks than the IPI itself and must be considered.

In conclusion, short IPIs were protective for women and neutral for neonates, while long intervals were associated with increased maternal and neonatal morbidity. The lowest risk was seen at 6–11 months’ interval. In a high-resource setting, the relevance of the policy formulated by the WHO of a recommended 24 months’ IPI may be questioned and women may choose shorter IPI without increased risks.

## Supplementary Information

Supplementary Information.

## Data Availability

This paper uses data from Statistics Sweden and the Swedish National Board of Health and Welfare. Because the data contain sensitive information on individuals, the Swedish law requires that users of the data obtain permissionfrom the Swedish Ethical Review Authority.
