# Supplementary material for: Interpregnancy interval and maternal and neonatal morbidity: a nationwide cohort study
Source: Sci Rep. 2022 Oct 18;12:17402. doi: 10.1038/s41598-022-22290-1 (PMC9579163; doi:10.1038/s41598-022-22290-1)
Supplement: Supplementary file 1 — Supplementary Information. [file 41598_2022_22290_MOESM1_ESM.docx]

**Supporting information**

**Table S1. Definitions of maternal morbidity.**

|  | ICD-10 codes, KVÅ codes or variable defined by the Swedish Medical Birth Register (SMBR) |
| --- | --- |
| Severe morbidity |  |
| Maternal death | O95 O959 O96 O960 O961 O969 O97 O970 O971 O979 |
| Sepsis | O859 R572 A41 |
| Eclampsia | O15 O151 O152 O159 |
| Non-elective hysterectomy | MCA33 MCA30 LCD00 LCD96 ZXD00 |
| Other surgical interventions such as uterine compression sutures (B-Lynch), uterine artery ligation, embolization, internal iliac artery ligation or intrauterine balloon tamponade | MBB10 DP015 MWE |
| Transfusion | DR029 DR036 V9209 |
| Maternal venous thromboembolism | O871 O873 O878 O880 O881 O882 O883 O888 |
| Uterine rupture and other major obstetric injuries (inversion, cervical laceration, obstetric hematoma) | O710 O711 O712 O713 O714 O715 O716 O717 O718 O719 |
| Intrapartum cesarean section (2^nd^ birth) | SMBR |
| 3^rd^ and 4^th^ degree perineal laceration | SMBR plus O702A O702B O702C O702D O702E O702X O703 MBC33 |
|  |  |
| Moderate morbidity |  |
| Postpartum hemorrhage (>1000 ml) | O678 O720 O721A O721B O721X O722 O723 |
| Curettage or vacuum aspiration after delivery | MBA00 MBA03 MBB10 |
| Preeclampsia/gestational hypertension | O139 O14 O140 O141 O141A O141B O141X O142 O149 |
| Diabetes, gestational | O244 O244A O244B |
| Chorioamnionitis | O411 R572 |
| Wound infection | O860 |
| Urinary tract infection, pyelonephritis | O862 O863 |
| Endometritis | O859 |
| Prelabor cesarean section (2^nd^ birth) | SMBR |
| Forceps | SMBR |
| VE | SMBR |
| Episiotomy | SMBR |

**Table S2. Definitions of neonatal morbidity.**

|  | **ICD-10 codes, KVÅ codes or variable defined by the Swedish Medical Birth Register (SMBR)** |
| --- | --- |
| **Severe neonatal morbidity** |  |
| Stillbirth | SMBR |
| Cardiorespiratory resuscitation (intubation, ventilation, correction of acidosis, heart compressions) | V9335 DG017 DG018 DM004 DG010 V9231 DF017 V9203 V9337 DF012 |
| Mechanical ventilation within first 72 hours | DG021 DG022 DG002 V9201 |
| Hypoxic ischemic encephalopathy 2-3 | P91 P910 P911 P912 P913 P915 P916 P916A P916B P916C P916X P917 P918 P919 |
| Neonatal convulsions | P90 P909 P909A P909B P909C |
| Therapeutic hypothermia | DV034 |
| Umbilical artery pH <7.00 | P21 P210 P211 P211A P211B P219 |
| Apgar score <4 at 5 minutes | SMBR |
| Meconium aspiration syndrome | P240 P220 P221 P228 P229 P22 |
| Hypoglycemia requiring IV therapy | P702 P703 P704 P704A P704B P708 |
| Intracranial hemorrhage | P10 P100 P101 P102 P103 P102 P103 P104 P108 P109 P52 P520 P521 P523 P524 P525 P526 P528 P529 P11 P110 P111 P112 P113 P119 |
| Birth trauma (fractures, neurologic injury, retinal hemorrhage or facial nerve palsy) | P11 P110 P111 P112 P113 P114 P115 P119 P13 P130 P131 P132 P133 P134 P138 P139 P148 P149 H356 |
| Cerebral palsy | G80 G800 G801 G801A G801B G801X G802 G803 G803A G803B G803X G804 G804A G804W G808 G808B G808C G808W G809 |
| Sepsis | P36 P360 P361 P362 P363 P364 P365 P368 P369 |
| Pneumonia | P23 P230 P231 P232 P233 P234 P234 P235 P236 P238 P239 |
| Birthweight <1500 g | SMBR |
| Preterm birth <32 weeks | SMBR |
|  |  |
| **Moderate neonatal morbidity** |  |
| Apgar score 4-6 at 5 minutes | SMBR |
| Obstetric brachial plexus injury | P140 P141 P143 P148 P149 |
| Cephalo-/subgaleal hematoma | P120 P122 P128 P129 |
| Neonatal jaundice requiring phototherapy or exchange transfusion | P570 P578 P579 P58 P580 P581 P582 P583 P584 P588 P589 P59 P590 P591 P592 P593 P598 P59 P599 DR050 |
| Macrosomia (≥4500 g) | SMBR |
| CPAP or high flow nasal cannula | DG001 V9375 |
| Birthweight 1500-2500 g | SMBR |
| Preterm birth 32-36 weeks | SMBR |

**Table S3. Association between interpregnancy interval and composite maternal morbidity.**

|  | **Severe maternal morbidity** | | | | **Moderate maternal morbidity** | | | |
| --- | --- | --- | --- | --- | --- | --- | --- | --- |
|  | Unadjusted | | Adjusted^*^ | | Unadjusted | | Adjusted^*^ | |
| Months | OR | 95% CI | aOR | 95% CI | OR | 95% CI | aOR | 95% CI |
| <6 | 0.90 | [0.80-1.01] | 0.95 | [0.84-1.06] | 0.79 | [0.74-0.85] | 0.86 | [0.80-0.93] |
| 6-11 | 0.86 | [0.81-0.91] | 0.89 | [0.84-0.95] | 0.82 | [0.79-0.85] | 0.85 | [0.82-0.89] |
| 12-17 | 0.89 | [0.84-0.94] | 0.91 | [0.86-0.96] | 0.86 | [0.84-0.89] | 0.88 | [0.85-0.91] |
| 18-23 | 0.95 | [0.90-1.01] | 0.97 | [0.92-1.02] | 0.95 | [0.92-0.98] | 0.96 | [0.93-1.00] |
| 24-29 | Reference (1.0) | | | | Reference (1.0) | | | |
| 30-35 | 1.07 | [1.01-1.14] | 1.07 | [1.00-1.13] | 1.09 | [1.05-1.13] | 1.08 | [1.04-1.13] |
| 36-41 | 1.11 | [1.04-1.18] | 1.10 | [1.02-1.17] | 1.15 | [1.11-1.20] | 1.14 | [1.09-1.19] |
| 42-47 | 1.17 | [1.08-1.26] | 1.15 | [1.07-1.24] | 1.18 | [1.13-1.24] | 1.17 | [1.12-1.23] |
| 48-53 | 1.19 | [1.09-1.29] | 1.15 | [1.06-1.26] | 1.26 | [1.20-1.33] | 1.24 | [1.17-1.31] |
| 54-59 | 1.19 | [1.08-1.31] | 1.15 | [1.04-1.27] | 1.28 | [1.21-1.36] | 1.26 | [1.19-1.35] |

OR=odds ratio, aOR=adjusted odds ratio, 95% confidence intervals (CI) in brackets.

*^*^ Adjustment was made for educational attainment, body mass index, age, born in Sweden or not, smoking, in vitro fertilization, less than 5 prenatal visits, sickness benefits or hospitalization prior to the first birth, and severe and moderate maternal and neonatal morbidity in the first birth.*

**Table S4. Association between interpregnancy interval and composite neonatal morbidity.**

|  | **Severe neonatal morbidity** | | | | **Moderate neonatal morbidity** | | | |
| --- | --- | --- | --- | --- | --- | --- | --- | --- |
|  | Unadjusted | | Adjusted^*^ | | Unadjusted | | Adjusted^*^ | |
| Months | OR | 95% CI | aOR | 95% CI | OR | 95% CI | aOR | 95% CI |
| <6 | 1.14 | [1.02-1.28] | 0.93 | [0.82-1.05] | 1.20 | [1.11-1.29] | 1.01 | [0.93-1.10] |
| 6-11 | 0.96 | [0.89-1.02] | 0.90 | [0.84-0.96] | 1.01 | [0.97-1.06] | 0.97 | [0.92-1.01] |
| 12-17 | 0.95 | [0.90-1.01] | 0.94 | [0.88-0.99] | 0.98 | [0.95-1.02] | 0.97 | [0.94-1.01] |
| 18-23 | 0.97 | [0.91-1.03] | 0.97 | [0.92-1.03] | 1.01 | [0.97-1.05] | 1.01 | [0.97-1.06] |
| 24-29 | Reference (1.0) | | | | Reference (1.0) | | | |
| 30-35 | 1.11 | [1.04-1.18] | 1.10 | [1.03-1.18] | 1.11 | [1.06-1.16] | 1.10 | [1.05-1.15] |
| 36-41 | 1.12 | [1.04-1.21] | 1.09 | [1.01-1.18] | 1.11 | [1.05-1.16] | 1.08 | [1.03-1.14] |
| 42-47 | 1.18 | [1.08-1.28] | 1.15 | [1.06-1.25] | 1.18 | [1.11-1.25] | 1.15 | [1.09-1.22] |
| 48-53 | 1.29 | [1.17-1.41] | 1.22 | [1.11-1.34] | 1.21 | [1.14-1.29] | 1.16 | [1.08-1.24] |
| 54-59 | 1.33 | [1.20-1.47] | 1.26 | [1.13-1.41] | 1.20 | [1.11-1.29] | 1.15 | [1.06-1.24] |

OR=odds ratio, aOR=adjusted odds ratio, 95% confidence intervals in brackets.

^*^ Adjustment was made for educational attainment, body mass index, age, born in Sweden or not, smoking, in vitro fertilization, less than 5 prenatal visits, sickness benefits or hospitalization prior to the first birth, and severe and moderate maternal and neonatal morbidity in the first birth.

**Figure S1. Flow chart.**

Births in Sweden 1997-2017, n=1188381

Women with a first and second birth, n=611434

Women with multiple births, n=12323

Women with missing information on gestational age n=380. Women with longer IPI's than 60 months, n=34768 and women having their first child after 2012, n=88671

Final sample:

Women, n=327912

Neonates, n=655824

Women with stillbirth at first birth, n=2612

Mothers with a cesarean section at first birth, n=95112

Women with a cesarean section at first birth, n=95112

Women with missing values on covariates, n=49652, and women with contradicting information, n=4

Flow chart. The patient selection process using data from the Swedish Medical Birth Register.

**Figure S2. IPI distribution.**


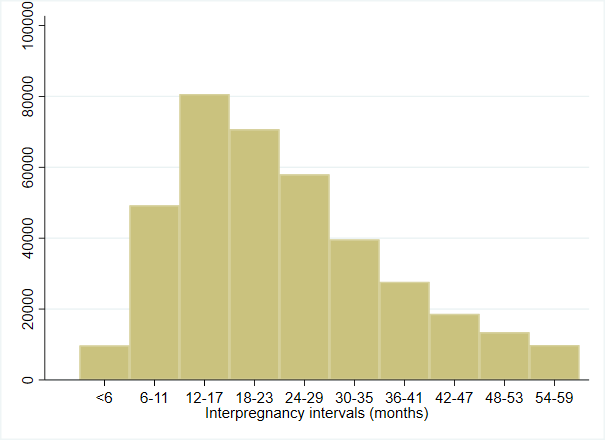


**Figure S3. Sensitivity analysis of the association between interpregnancy interval and maternal and neonatal morbidity including women with missing values.**

| a) Severe maternal morbidity | b) Moderate maternal morbidity |
| --- | --- |
| ***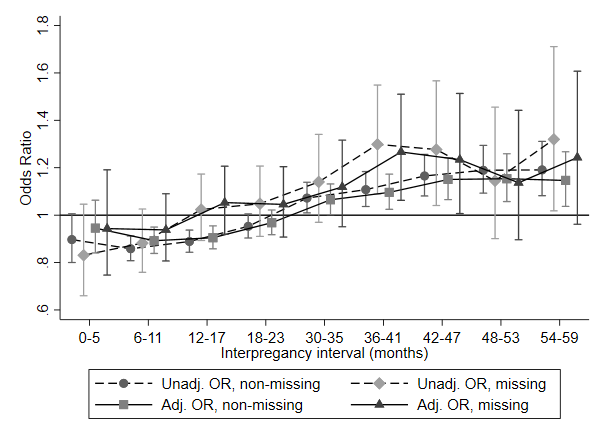*** | ***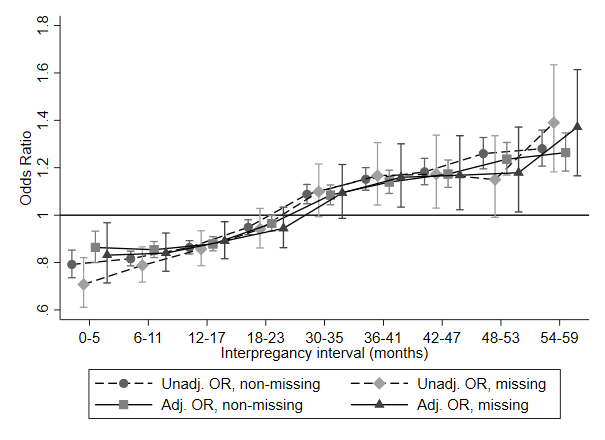*** |
| c) Severe neonatal morbidity | d) Moderate neonatal morbidity |
| 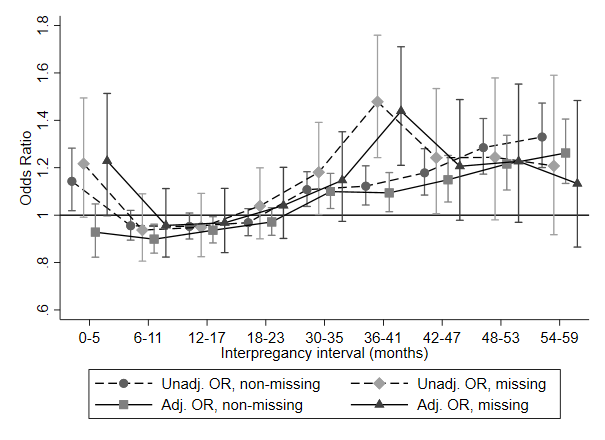 | 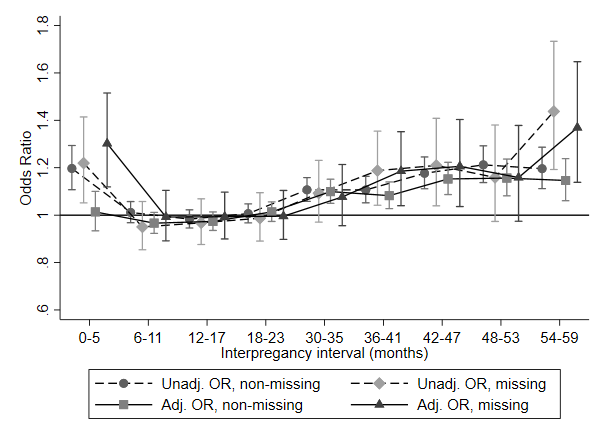 |

Unadj: unadjusted, Adj: adjusted, OR: odds ratio. The odds ratios are presented with 95% confidence intervals marked by the whiskers. 24-29 months are the reference interval. No difference in odds ratios (1) is marked with a solid line. Values below 1 represent a decreased risk and values above 1 represent an increased risk. Adjustment was made for severe and moderate maternal and neonatal morbidity in the first birth.
